# Supplementary material for: The causal role of circulating inflammatory markers in osteoporosis: a bidirectional Mendelian randomized study
Source: Front Immunol. 2024 Jul 18;15:1412298. doi: 10.3389/fimmu.2024.1412298 (PMC11291241; doi:10.3389/fimmu.2024.1412298)
Supplement: Supplementary file 1 [file DataSheet_1.zip › supplementary material/CIM-OSTEOPOROSIS /GCST90274820.csv_leave_one_out.pdf]

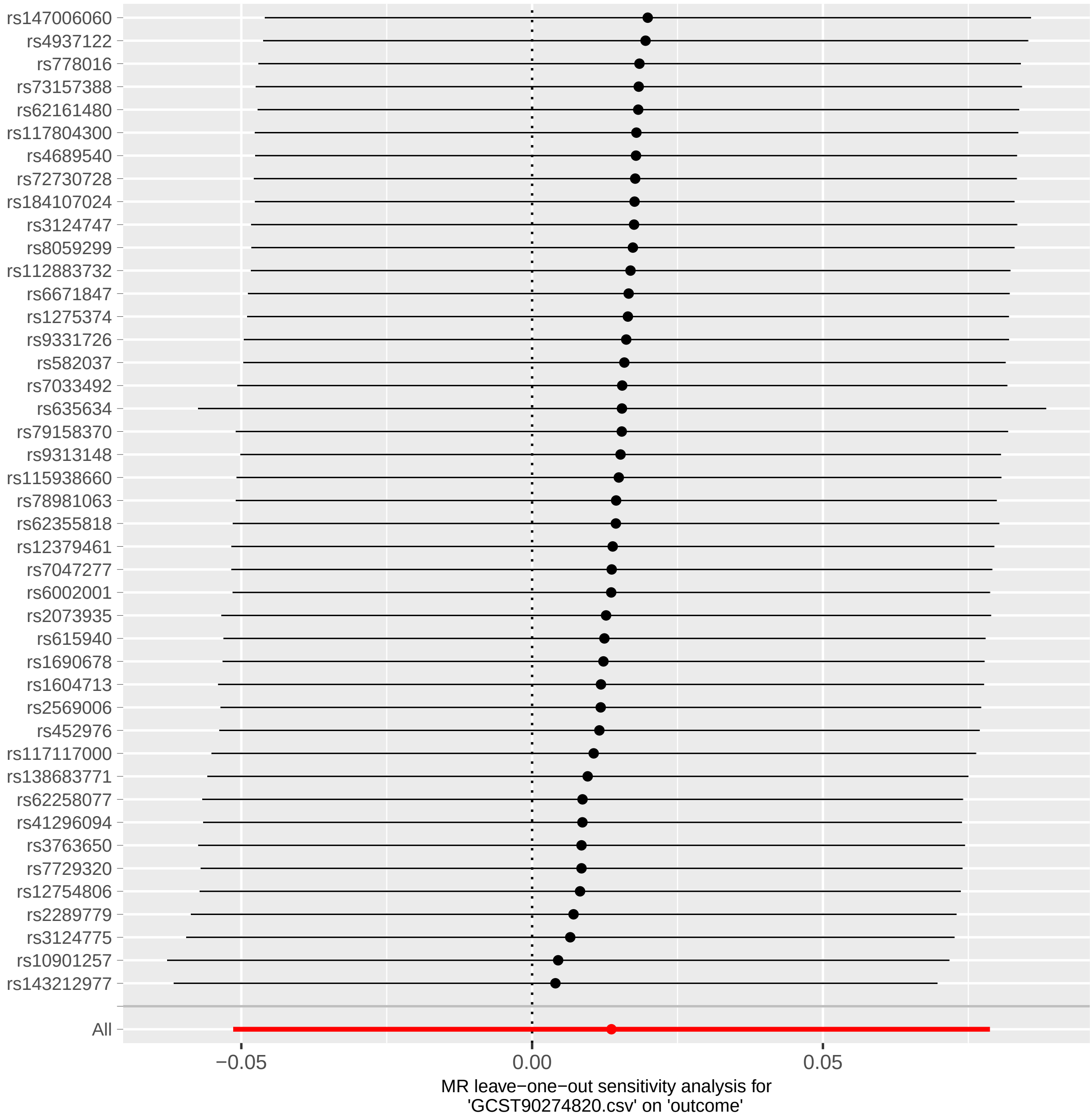
rs147006060
rs4937122
rs778016
rs73157388
rs62161480
rs117804300
rs4689540
rs72730728
rs184107024
rs3124747
rs8059299
rs112883732
rs6671847
rs1275374
rs9331726
rs582037
rs7033492
rs635634
rs79158370
rs9313148
rs115938660
rs78981063
rs62355818
rs12379461
rs7047277
rs6002001
rs2073935
rs615940
rs1690678
rs1604713
rs2569006
rs452976
rs117117000
rs138683771
rs62258077
rs41296094
rs3763650
rs7729320
rs12754806
rs2289779
rs3124775
rs10901257
rs143212977
All
−0.05
0.00
0.05
MR leave−one−out sensitivity analysis for
'GCST90274820.csv' on 'outcome'
